# Identification of TRAIL-inducing compounds highlights small molecule ONC201/TIC10 as a unique anti-cancer agent that activates the TRAIL pathway

**DOI:** 10.1186/s12943-015-0346-9

**Published:** 2015-05-01

**Authors:** Joshua E Allen, Gabriel Krigsfeld, Luv Patel, Patrick A Mayes, David T Dicker, Gen Sheng Wu, Wafik S El-Deiry

**Affiliations:** Departments of Medicine, Genetics, and Pharmacology, Laboratory of Molecular Oncology and Cell Cycle Regulation, University of Pennsylvania School of Medicine, Philadelphia, 19104 PA USA; Current affiliation: Oncoceutics, Inc., Hummelstown, PA USA; Department of Medical Oncology and Molecular Therapeutics Program, Laboratory of Translational Oncology and Experimental Cancer Therapeutics, Fox Chase Cancer Center, Philadelphia, 19111 PA USA; Department of Pathology, Program in Molecular Biology and Genetics, Karmanos Cancer Institute, Wayne State University School of Medicine, Detroit, MI 48201 USA

**Keywords:** ONC201, TIC10, TRAIL, TRAIL-inducing compound, TNF-related apoptosis-inducing ligand, DR5, Foxo3a, Gene regulation

## Abstract

**Background:**

We previously reported the identification of ONC201/TIC10, a novel small molecule inducer of the human TRAIL gene that improves efficacy-limiting properties of recombinant TRAIL and is in clinical trials in advanced cancers based on its promising safety and antitumor efficacy in several preclinical models.

**Methods:**

We performed a high throughput luciferase reporter screen using the NCI Diversity Set II to identify TRAIL-inducing compounds.

**Results:**

Small molecule-mediated induction of TRAIL reporter activity was relatively modest and the majority of the hit compounds induced low levels of TRAIL upregulation. Among the candidate TRAIL-inducing compounds, TIC9 and ONC201/TIC10 induced sustained TRAIL upregulation and apoptosis in tumor cells in vitro and in vivo. However, ONC201/TIC10 potentiated tumor cell death while sparing normal cells, unlike TIC9, and lacked genotoxicity in normal fibroblasts. Investigating the effects of TRAIL-inducing compounds on cell signaling pathways revealed that TIC9 and ONC201/TIC10, which are the most potent inducers of cell death, exclusively activate Foxo3a through inactivation of Akt/ERK to upregulate TRAIL and its pro-apoptotic death receptor DR5.

**Conclusion:**

These studies reveal the selective activity of ONC201/TIC10 that led to its selection as a lead compound for this novel class of antitumor agents and suggest that ONC201/TIC10 is a unique inducer of the TRAIL pathway through its concomitant regulation of the TRAIL ligand and its death receptor DR5.

## Introduction

TRAIL is an endogenous protein that induces fulminant tumor-specific apoptosis through binding to death receptors DR4 or DR5 expressed in human tumor cells [1]. TRAIL has received considerable attention since the gene was first cloned because of its therapeutic potential as a drug target for human cancer due to its ability to distinguish tumor from normal cells. TRAIL is naturally expressed in a several human tissues and membrane-bound TRAIL is also conditionally expressed in some immune cells following cytokine stimulation [2-6]. Through its expression in such cells, TRAIL plays a direct role in tumor suppression during immune surveillance though this anticancer mechanism is lost during the disease progression.

The ability of TRAIL to initiate apoptosis selectively in cancer cells has led to clinical trials with novel agents that engage the TRAIL pathway, which includes recombinant TRAIL and TRAIL-agonist antibodies that target DR4 or DR5 [7-13]. TRAIL-based experimental therapies have exhibited promising preclinical activity and safety in early phase clinical trials [14]. Nevertheless, these investigational therapies did not prove sufficiently effective in clinical trials and the clinical development of recombinant TRAIL has been halted. While the reasons for clinical failure are not entirely clear, we and others have highlighted several undesirable drug properties that may hinder the efficacy of recombinant TRAIL such as serum half-life, stability, and/or biodistribution.

Several experimental efforts to improve the efficacy of TRAIL-targeted therapies have been reported. Recombinant TRAIL mutants that are remarkably more stable have been identified [15], as well as variants that contain leucine or isoleucine zippers to facilitate trimerization of the soluble ligand, since receptor-bound TRAIL is trimeric [16,17]. We previously reported a novel class of DR4-targeted proteins called DR4 Atrimers that are engineered to mimic the conformation of trimeric TRAIL bound to DR4 using a stable tetranectin scaffold [18]. Mesenchymal stem cells overexpressing TRAIL have been described in preclinical studies that improve the biodistribution of TRAIL to enable activity against glioma since the available TRAIL-based therapies do not cross the blood–brain barrier [19]. In vitro characterization and structure-activity relationships of small molecules that induce DR5 clustering and activation have also be reported [20].

TRAIL is a robust and selective tumor suppressor that offers itself as an attractive natural drug target to restore anti-tumor immunity. We hypothesized that upregulation of TRAIL expression by a small molecule would lead to a potent and novel anti-tumor mechanism by improving suboptimal drug properties of recombinant TRAIL. Regulation of the TRAIL gene has been described for several transcription factors [21], most of which are tumor suppressors such as p53 [22], and Foxo3a [23]. We explicitly selected for TRAIL-inducing compounds that upregulate TRAIL gene transcription using a mechanism that does not rely on p53 due to its frequent inactivation in late stage cancers that causes resistance to many standard-of-care therapies [24]. To identify small molecule p53-independent inducers of the human TRAIL gene we conducted a small molecule library screen using the NCI Diversity Set II. The screen was conducted in HCT116 cells lacking the Bax gene, which renders TRAIL resistance to allow for assay readout [25], and stably expressing a luciferase reporter of the human TRAIL gene promoter. Here we describe the preclinical studies that led to the selection of ONC201/TIC10 as the lead TRAIL-inducing compound that we previously reported as a novel and potent antitumor agent and has entered phase I clinical trials in advanced cancers [26].

## Results

### Screening for small molecules that induce TRAIL gene promoter activity

**Figure 1:**
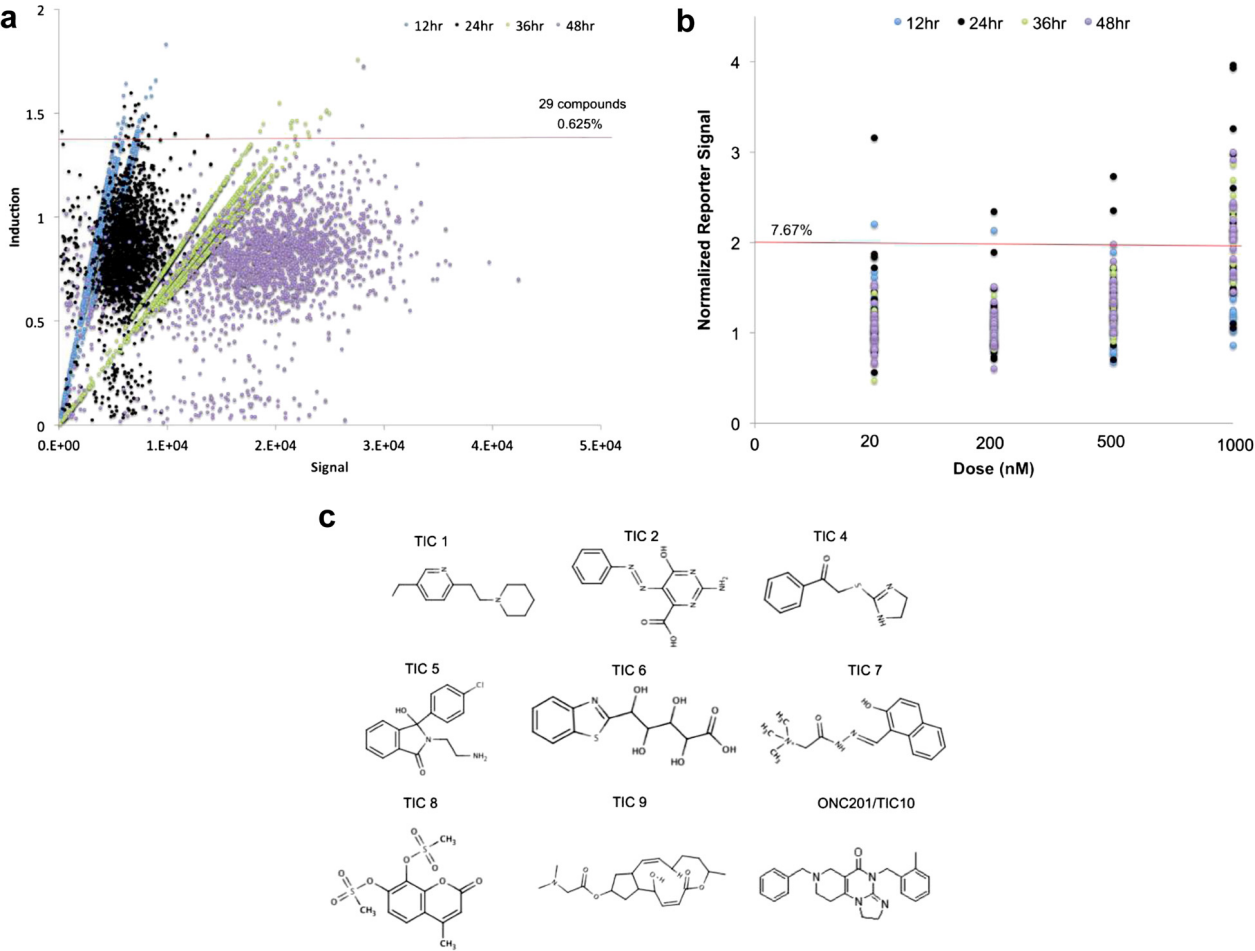
**Identification of TRAIL-inducing compounds by high-throughput luciferase reporter screen. (a)** Plot of reporter induction versus signal for screen conducted with the NCI Diversity Set II at 1 μM in HCT116 Bax^−/−^ cells that stably express a luciferase gene reporter construct under transcriptional control of the first 504 base pairs of the human TRAIL gene promoter. Induction is expressed as luminescence relative to DMSO-treated cells. The horizontal red line represents the cutoff for selection. **(b)** Validation of reporter induction for candidate screen hits in HCT116 Bax^−/−^ cells at multiple doses and time points. Reporter signal is expressed relative to DMSO-treated cells and normalized to cell viability. The horizontal red line represents the cutoff for selection. Data represent an average of three replicates. The horizontal red line represents the cutoff for selection. **(C)** Molecular structures of the selected TRAIL-inducing compounds.

We transiently transfected HCT116 Bax-null cells with a luciferase gene reporter construct under transcriptional control of the first 504 base pairs of the human TRAIL gene promoter and selected for clones with stable expression. The NCI Diversity Set II was tested at 20 nM, 200 nM, 500 nM, and 1 μM using this cell-based reporter system at 12, 24, 36, and 48 hours post-treatment. Overall the small molecule library resulted in relatively modest changes in the TRAIL gene reporter activity, with most molecules causing a decrease in reporter activity due to cytotoxic effects or repression of the reporter (Figure 1a). We selected 29 compounds that induce >1.4-fold induction of reporter activity for further study. Normalizing for change in cell viability, we found that 10 of these 29 compounds were able to upregulate TRAIL reporter activity >2-fold under at least 2 of the tested conditions, with most of this activity at a dose of 1 μM (Figure 1b-c).

### TIC9 and ONC201/TIC10 induce TRAIL and apoptosis in vivo

**Figure 2:**
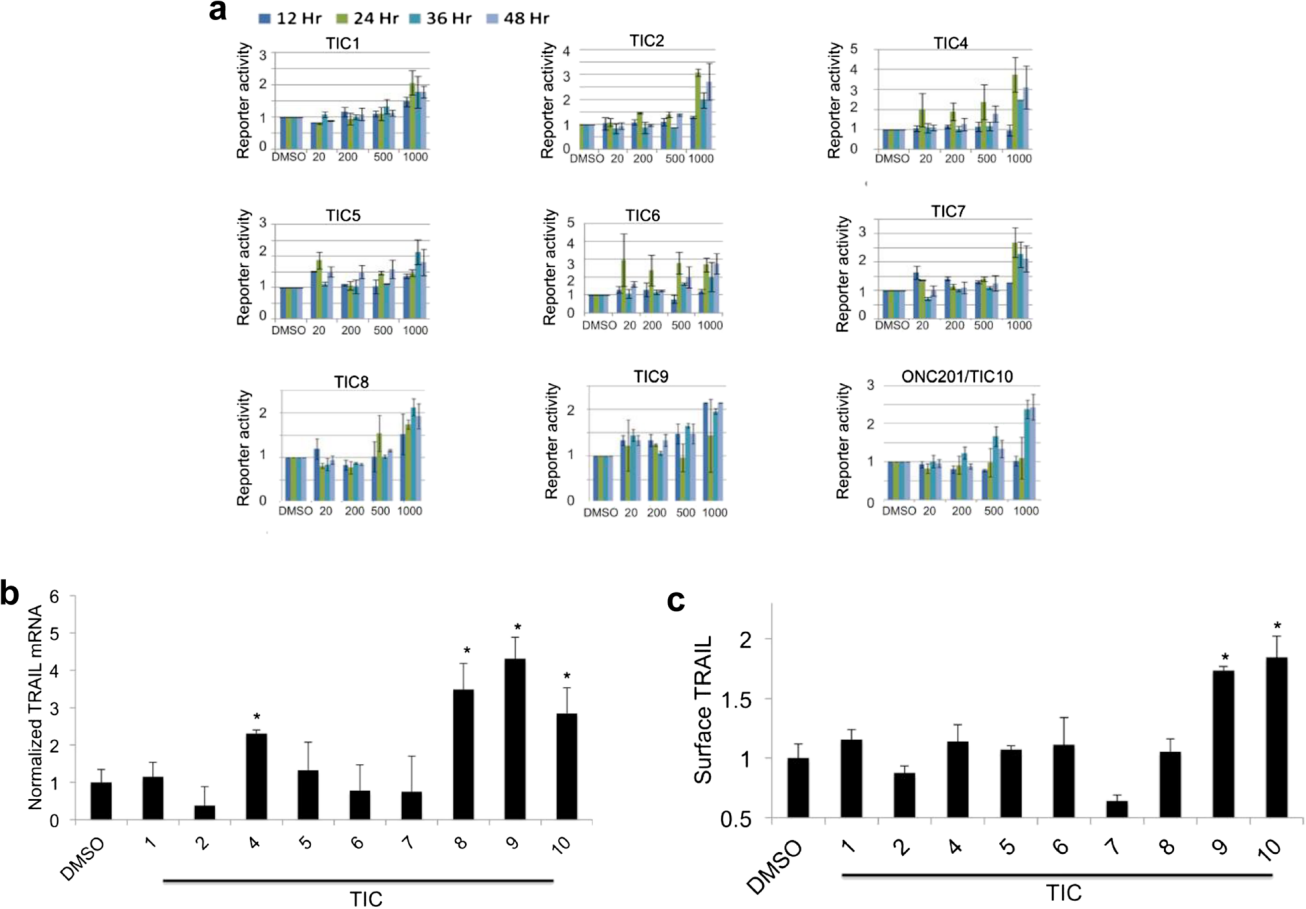
**TIC9 and ONC201/TIC10 induce TRAIL gene transcription and surface TRAIL upregulation in tumor cells in vitro. (a)** Reporter activity in HCT116 Bax^−/−^ cells that stably express a luciferase gene reporter construct under transcriptional control of the first 504 base pairs of the human TRAIL gene promoter (n = 3). Reporter signal is expressed relative to DMSO-treated cells and normalized to cell viability. **(b)** RT-qPCR analysis of TRAIL gene transcription in response to TRAIL-inducing compounds (TICs) (5 μM) in HCT116 p53^−/−^ cells (n = 4, 48 hr). **(c)** Flow cytometry analysis of surface TRAIL in response to TICs (5 μM) in HCT116 p53^−/−^ cells (n = 3, 72 hr). *P < .05 by student’s two-tailed *t* test. Error bars represent standard deviation.

Nine of these 10 compounds that induce TRAIL gene promoter reporter activity were selected for further characterization, as TIC3 was unavailable at the time of study (Figure 1d). Interestingly TIC9 is breflate, the pro-drug of the small molecule brefeldin A that is a classic ER stress-inducer. In general, these 9 small molecules stimulated TRAIL gene promoter reporter activity in a dose-dependent manner and at time points ≥24 hours post-treatment (Figure 2a). RT-qPCR analysis of p53-deficient HCT116 cells revealed that TIC4, TIC8, TIC9, and ONC201/TIC10 were capable of upregulating TRAIL messenger RNA levels under the tested conditions in a p53-deficient background (Figure 2b). Next we assessed the capability of these molecules to upregulate TRAIL on the surface of tumor cells. TIC9 and ONC201/TIC10 were the only compounds capable of upregulating TRAIL protein at the surface of HCT116 cells under the tested conditions (Figure 2c).

**Figure 3:**
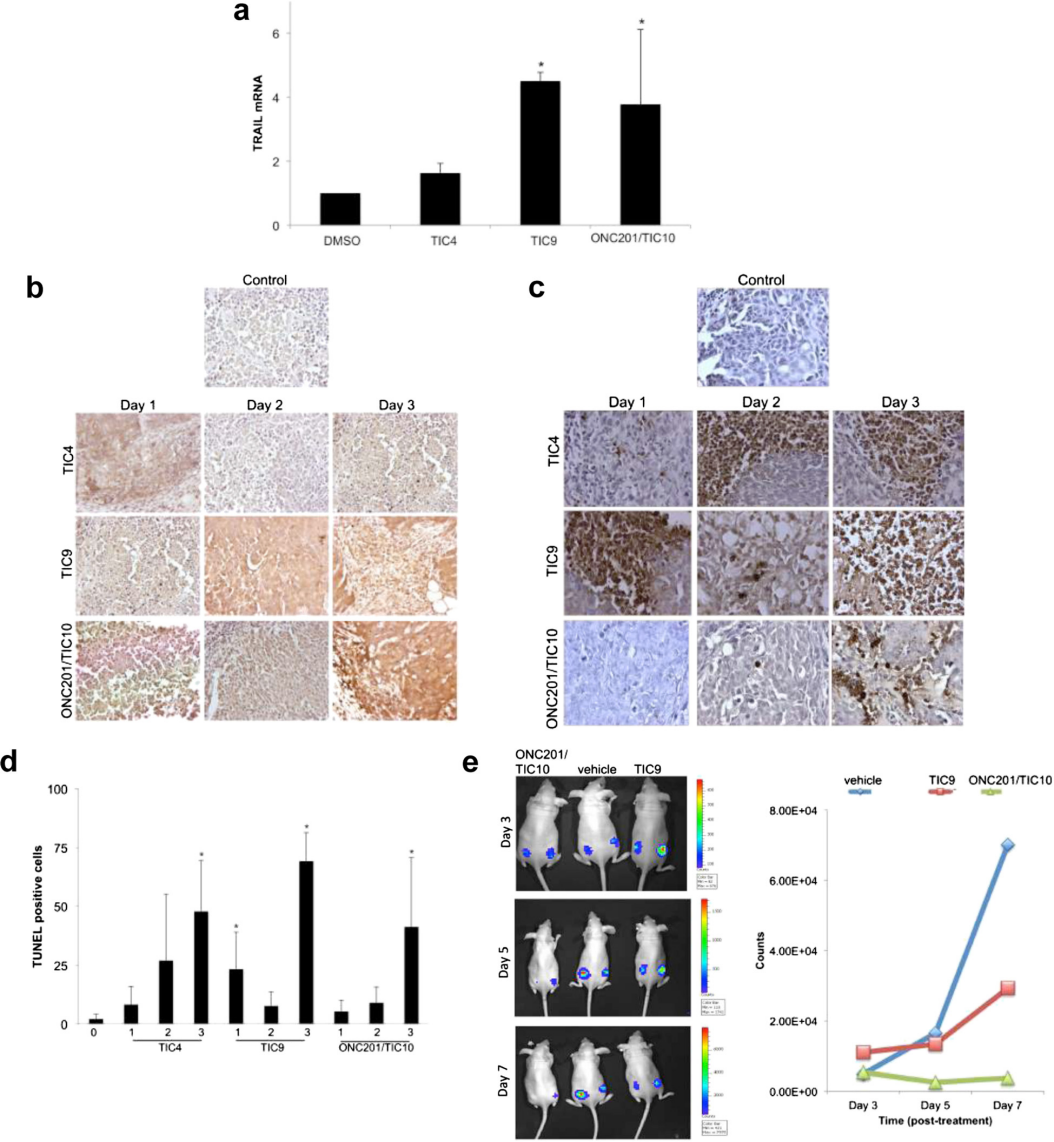
**TIC4, TIC9, and ONC201/TIC10 upregulate TRAIL in vivo. (a)** RT-qPCR analysis of HCT116 p53^−/−^ tumor xenograft in athymic nude mice harvested 2 days following a single intravenous dose of DMSO, TIC4, TIC9, or ONC201/TIC10 (25 mg/kg). *P < .05 by student’s two-tailed *t* test. **(b)** IHC analysis of TRAIL protein levels in HCT116 p53^−/−^ tumor xenograft in athymic nude mice harvested following a single intravenous dose of DMSO, TIC4, TIC9, or ONC201/TIC10 (25 mg/kg). **(c)** Exemplary images and **(d)** quantification of TUNEL staining in TIC-treated xenograft tumors described in **(a)**. **(e)** Bioluminescent imaging of luciferase-infected HCT116 subcutaneous xenografts following a single intraperitoneal dose of TIC9 and ONC201/TIC10 (100 mg/kg) on day 0. Quantification of tumor signals shown in right panel (n = 6). Error bars represent standard deviation.

The ability of TIC9 and ONC201/TIC10 to induce TRAIL in vivo was assessed in subcutaneous xenografts of HCT116 cells following a single intraperitoneal dose. Messenger RNA harvested from tumor xenografts found that TRAIL transcript levels were significantly elevated by both TIC9 and ONC201/TIC10 compared to vehicle treatment or TIC4 treatment, which was used as a negative control comparator (Figure 3a). Immunohistochemical analysis of treated tumor xenografts indicated that TIC9 and ONC201/TIC10 also elevate TRAIL protein levels in vivo (Figure 3b). Assessment of apoptosis in treated tumor xenografts revealed that TIC9 induces cell death as soon as the first day following treatment in contrast with ONC201/TIC10, which induces high levels of apoptosis at day 3 (Figure 3c-d). Interestingly, TIC4 also induces significant levels of apoptosis in a time-dependent manner that suggests the molecule may have TRAIL-independent pro-apoptotic effects on tumors in vivo. Based on the ability of TIC9 and ONC201/TIC10 to induce TRAIL and apoptosis in tumor xenografts, we performed an in vivo study to assess the antitumor activity of these two small molecules by bioluminescent imaging of luciferase-infected HCT116 subcutaneous xenografts. Administration of a single dose of ONC201/TIC10 potently inhibited the tumor signal following administration of a single dose (Figure 3e). TIC9 also inhibited the bioluminescent signal of the tumor compared to vehicle-treated xenografts but was inferior to ONC201/TIC10 under the tested conditions.

### Selection of ONC201/TIC10 as a lead TRAIL-inducing compound

**Figure 4:**
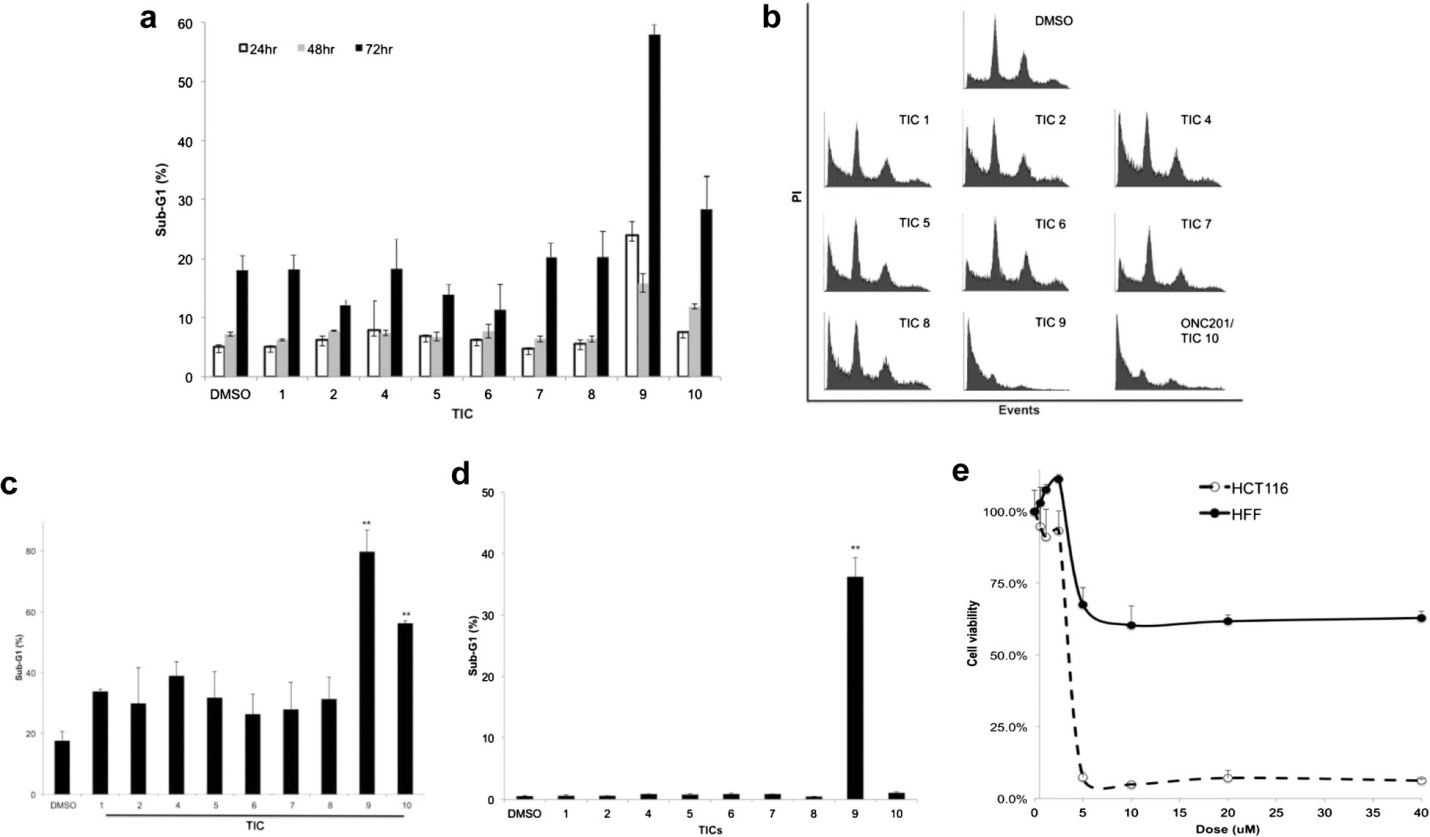
**TIC9 and ONC201/TIC10 induce tumor cell death and ONC201/TIC10-induced cell death is tumor-selective. (a)** Sub-G1 DNA content of HCT116 p53^−/−^ cells treated with TICs for indicated time periods (1 μM, n = 3). **(b)** Cell cycle profiles of HCT116 p53^−/−^ cells treated with TICs (5 μM, 72 hr). **(c)** Sub-G1 DNA content of HCT116 p53^−/−^ cells treated with TICs (72 hrs, 5 μM, n = 3). **(d)** Sub-G1 DNA content of HFF normal human fibroblasts treated with TICs (72 hr, 5 μM, n = 3). **(e)** Cell viability assay of HCT116 or HFF cells treated with ONC201/TIC10 at the indicated doses (72 hr, n = 3). Error bars represent standard deviation.

A time-course analysis of cell death in HCT116 cells indicated that TIC9 and ONC201/TIC10 were exclusively capable of inducing cell death. In accordance with our other studies, ONC201/TIC10 induced a delayed and modest amount of cell death that was apparent at 72 hours post-treatment with a 1 μM dose (Figure 4a-b). Interestingly, TIC4 did not induce tumor cell death under these conditions despite the observation that the molecule induced apoptosis in vivo. Together, these observations suggest that TIC4 may have TRAIL-independent apoptotic activity that depends on physiological factors not present in vitro or requires higher doses. Assessing slightly higher doses of TRAIL-inducing compounds in the low micromolar range revealed that both TIC9 and ONC201/TIC10 are capable of inducing high levels of Sub-G1 DNA content in p53-deficent human tumor cells (Figure 4c). Assaying the effects of the same cytotoxic dose on normal human fibroblasts revealed that TIC9 also induced significant levels of cell death in these cells (Figure 4d). However ONC201/TIC10 did not induce any appreciable levels of cell death in normal cells under the same conditions that were cytotoxic to tumor cells, suggesting a favorable therapeutic window.

**Figure 5:**
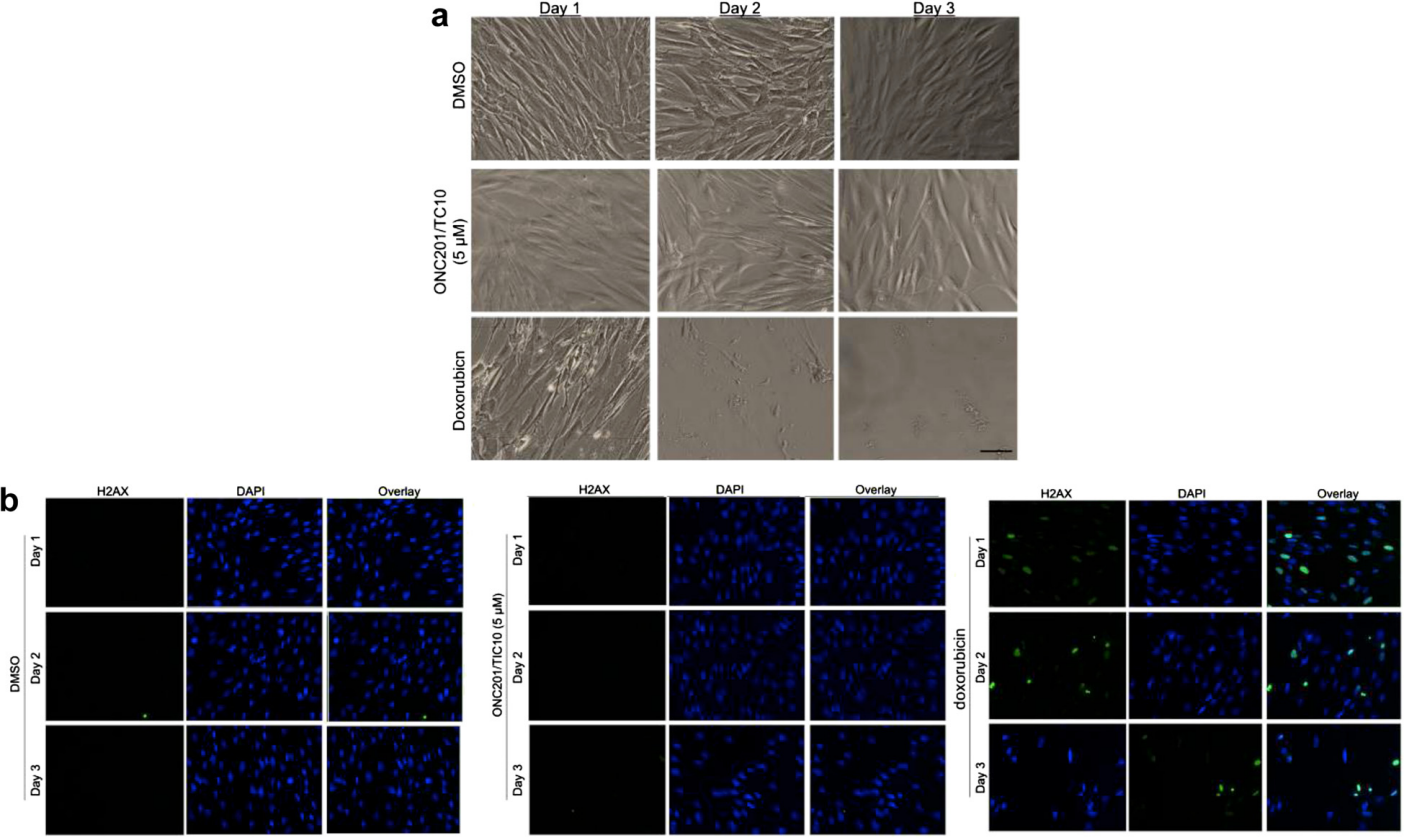
**ONC201/TIC10 is not genotoxic to normal human fibroblasts. (a)** Bright field microscopy images of HFF fibroblasts following 72 hr incubation with DMSO, ONC201/TIC10, or doxorubicin (1 μM) as indicated. **(b)** Immunofluorescence imaging of gamma H2AX (green) and nuclear counterstain (blue) in HFF fibroblasts treated as described in **(a)**.

**Figure 6:**
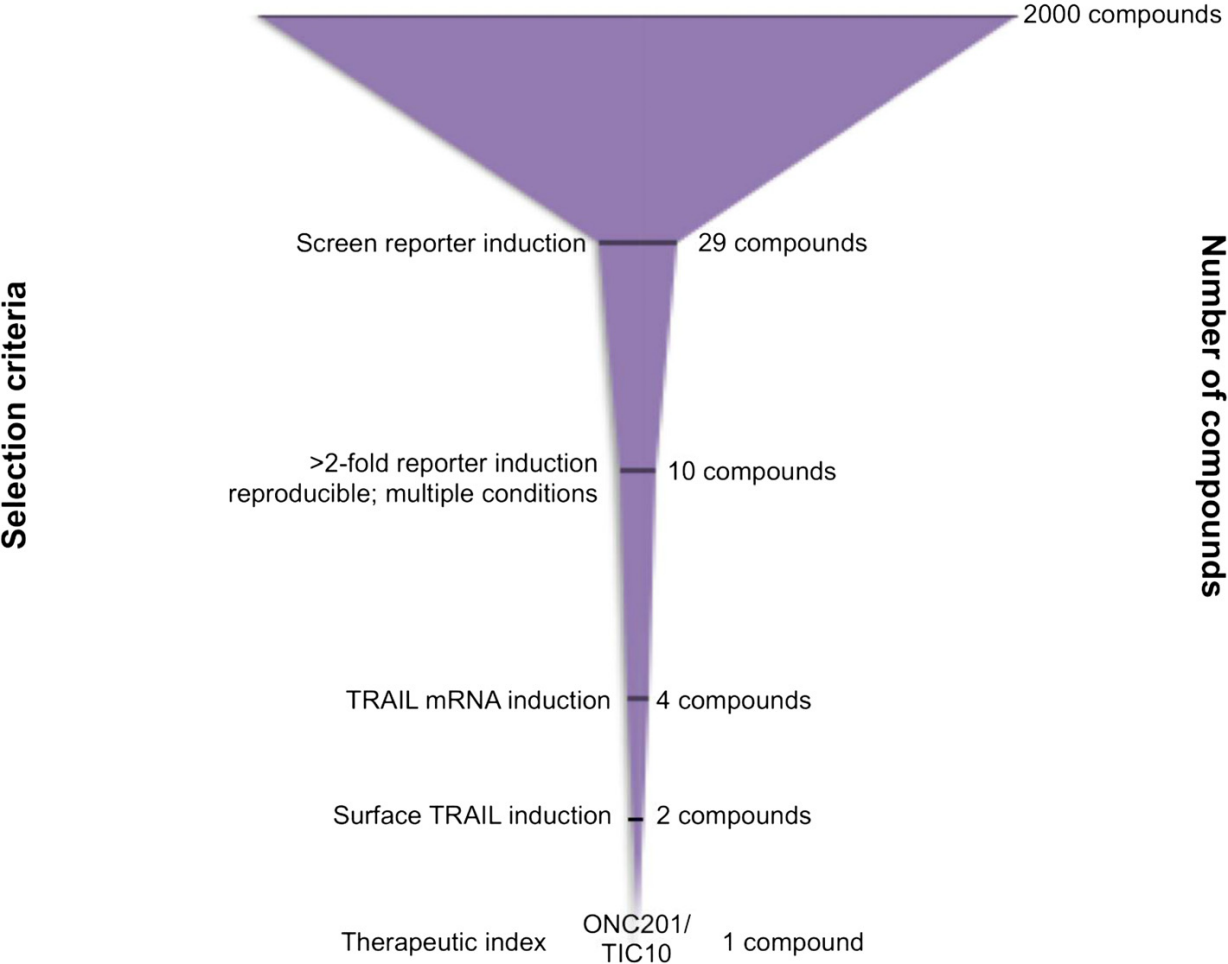
**Selection of ONC201/TIC10 as a lead TRAIL-inducing compound.** Prioritization assays are shown for the process of selecting ONC201/TIC10 as a lead compound.

Parallel cell viability assays with normal and tumor cells confirmed that ONC201/TIC10 has a wide therapeutic window. ONC201/TIC10 eliminated tumor cells in vitro with a dose–response relationship that was steep, saturable, and much more potent against tumor cells than normal cells (Figure 4e). Despite lack of induction of apoptosis in normal cells, ONC201/TIC10 appears to modestly inhibit the proliferation of normal cells at higher micromolar dose and an GI50 was not reached. We evaluated the potential effects of ONC201/TIC10 on normal cell morphology and genotoxicity by microscopy. Immunofluorescence experiments revealed that ONC201/TIC10 does not induce changes in the morphology or levels of gamma-H2AX in normal cells, unlike the DNA-damaging chemotherapy doxorubicin, despite long-term incubation with doses that are cytotoxic to tumor cells (Figure 5). A similar lack of genotoxicity or alteration of normal cell morphology was observed at lower doses of ONC201 as well (data not shown). Together these studies rationalize the selection of ONC201/TIC10 as a lead TRAIL-inducing compound that upregulates TRAIL gene transcription and protein levels, induces tumor-specific cell death, and is not cytotoxic or genotoxic to normal cells (Figure 6).

### Cytotoxic TRAIL-inducing compounds exclusively active Foxo3a

**Figure 7:**
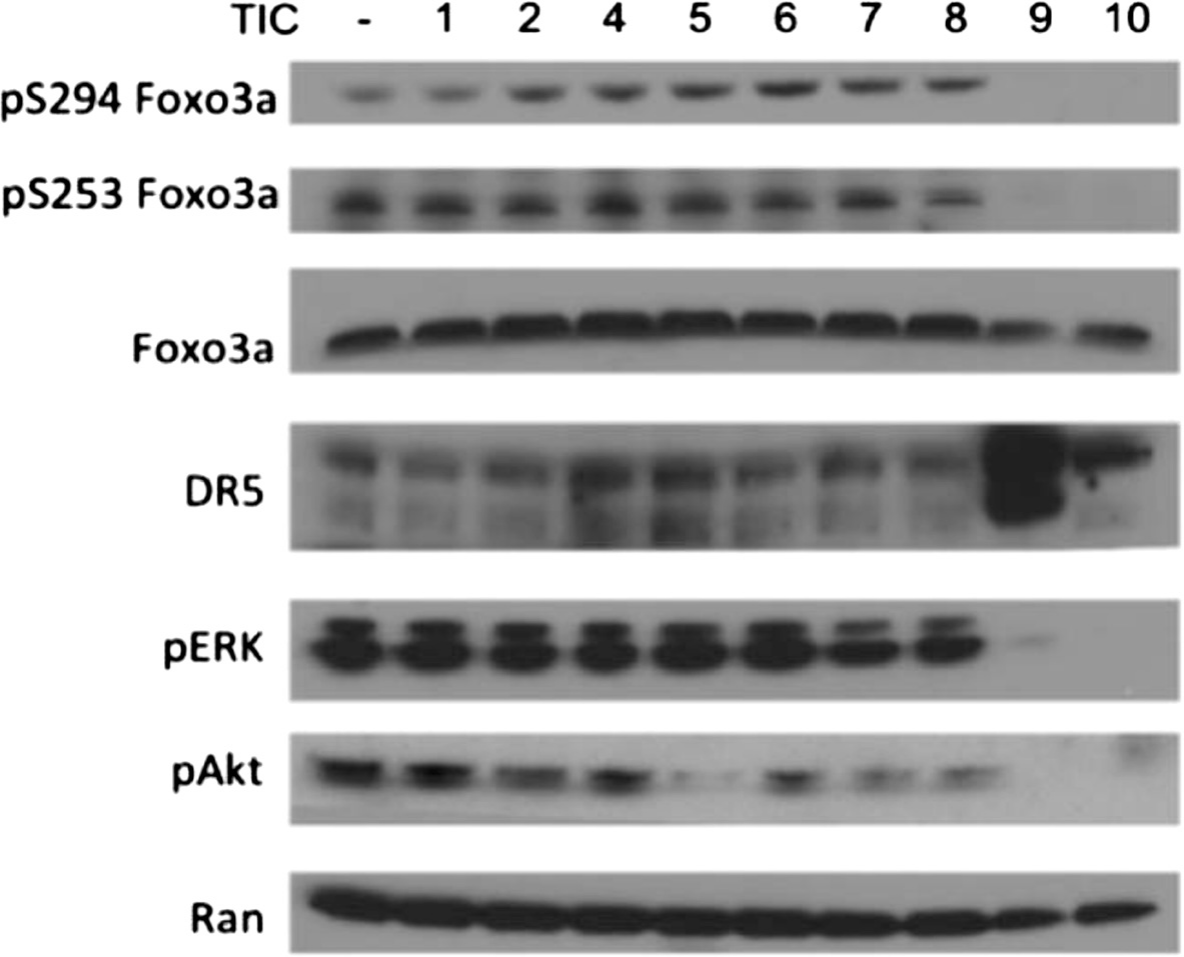
**ONC201/TIC10 and TIC9 exclusively affect Akt/ERK/Foxo3a.** Western blot analysis of HCT116 p53^−/−^ cells treated with TICs in HCT116 p53^−/−^ cells (5 μM, 60 hr).

Our previous studies with ONC201/TIC10 demonstrate that the small molecule induces TRAIL and TRAIL-mediated cell death through dephosphorylation and activation Foxo3a, which directly regulates TRAIL gene transcription at its gene promoter. Western blot analysis revealed that several of the TRAIL-inducing compounds reduced levels of phospho-Akt (Figure 7). Among the top TRAIL-inducing compounds, only TIC9 and ONC201/TIC10 inhibited phospho-ERK levels and caused the dephosphorylation of Foxo3a that is associated with its nuclear translocation and activation of target genes. Furthermore, TIC9 and ONC201/TIC10 were the only molecules that upregulated DR5, which is also a Foxo3a target gene that may contribute to the sensitivity of tumor cells to ONC201/TIC10-induced TRAIL. The observation that the exclusively cytotoxic TRAIL-inducing compounds also exclusively activate Foxo3a suggests that Foxo3a is a uniquely proapoptotic regulator of TRAIL-mediated apoptosis among other TRAIL gene regulators. Concomitant induction of TRAIL and DR5 by Foxo3a and perhaps other transcriptional mechanisms may explain the ability of ONC201/TIC10 to induce significant levels of apoptosis with modest induction of the ligand.

## Discussion

The magnitude and time points for kinetics of TRAIL induction observed in the screen for TRAIL-inducing compounds indicate that regulation of the TRAIL gene may be tightly controlled, perhaps due to its potent apoptotic potential. Chemical derivatives may be explored to improve TRAIL-inducing compounds in terms of potency and magnitude of TRAIL induction as well as potentially unveil structure-activity relationships. The observation that TIC9 and ONC201/TIC10 uniquely affect Akt- and ERK-mediated phosphorylation of Foxo3a is particularly interesting, given their exclusive ability to induce cancer cell death among the TRAIL-inducing compounds. While the two molecules are very diverse in structure and likely differ in other effects on cell signaling, this common effect suggests that Foxo3a may be an attractive transcriptional mechanism for inducing the TRAIL gene as an anticancer therapeutic mechanism.

While our first report of ONC201/TIC10 was in preparation, another report was published that described another novel class of small molecules that are capable of engaging the TRAIL death receptor pathway [20]. Identified in other efforts aiming to find small molecule Smac mimetics, Wang et al. reported a small molecule, bioymifi, that induces the clustering of DR5 and activation of its downstream apoptotic signaling. This novel therapeutic approach holds promise a new class of TRAIL-based agents, though its spectrum of activity and in vivo activity will need to be evaluated in future studies.

TRAIL-inducing compounds are a novel class of TRAIL-based therapy that engage tumors and the host system to upregulate TRAIL and potentially sensitize tumor cells to its pro-apoptotic activity through dual death receptor and ligand induction, as seen with TIC9 and ONC201/TIC10. Several chemotherapies have been reported to induce DR5 as a mechanism of tumor cell sensitization to TRAIL-mediated apoptosis [27]. Though p53 can also regulate the DR5 gene [28], both ONC201/TIC10 [26] and brefledin A [29], which TIC9 is a prodrug of, possess p53-independent anti-cancer activity that suggests DR5 induction occurs through other transcription factors. p53-independent induction of DR5 in tumor cells has been confirmed for ONC201 [26]. While both the TRAIL and DR5 gene promoters contain FOXO binding sites, future studies will explore the molecular mechanism of DR5 induction that may involve Foxo3a and/or other transcription factors. CHOP also has a binding site on the DR5 gene promoter that should be explored given recent reports of ONC201-induced activation of the integrated stress response [30].

These studies indicate that TIC9 was much more toxic to normal cells than ONC201/TIC10, which could be the result of a more pronounced DR5 induction by TIC9 that may carry over into normal cells unlike prior reports of ONC201/TIC10-induced DR5 in normal fibroblasts [26]. Clinical trials with ONC201/TIC10 have recently commenced in advanced cancers to evaluate its safety as a monoagent. The safety features of ONC201/TIC10 that were a key selection criteria in this study offer a range of clinical opportunities where genotoxic and toxic therapies are intractable. Furthermore, combination therapy may be facilitated by the absence of overlapping toxicities and broad synergy with approved anti-cancer compounds that was recently reported for ONC201/TIC10 [31]. Our studies cumulatively suggest that ONC201/TIC10 is a safe and effective antitumor agent that possesses a distinct mechanism of action that highlights the therapeutic potential of this novel class of anticancer drugs. This notion is further enhanced by its chemical structure, safety, and efficacy characteristics that are differentiated from other therapies used to treat cancer [32,33].

## Methods

### Cell culture and reagents

Cell lines were obtained from ATCC and cultured in ATCC-recommended media in a humidified incubator at 5% CO_2_ and 37°C. TICs were obtained from the NCI DTP, stored at −80°C, resuspended in DMSO, and maintained at −20°C for storage. The following compounds were ordered from the NCI DTP for follow up study: TIC1 (NSC 22819), TIC2 (NSC 96682), TIC4 (NSC 160319), TIC5 (NSC 320000), TIC6 (NSC 45741), TIC7 (NSC 64408), TIC8 (NSC 250826), TIC9 (NSC 656202), ONC201/TIC10 (NSC 350625).

### Cell death assays

For Sub-G1 DNA content analysis, cells were trypsinized at the indicated time points and fixed in 80% ethanol at 4°C for a minimum of 30 minutes. Fixed cells were then stained with propidium iodide in the presence of RNase and analyzed on an Epics Elite flow cytometer (Beckman Coulter). Cell viability was assessed using Cell TiterGlo (Promega) in 96-well plates using the manufacturer’s protocol.

### Western blot analysis

Cells were treated in log-phase growth, harvested by cell scraping, centrifuged, and lysed on ice for 2 hours with cell-lysis buffer. The supernatant was collected following centrifugation, and protein concentration was determined using the Bio-Rad protein assay (Bio-Rad Laboratories). Samples were electrophoresed under reducing conditions on NuPAGE 4-12% Bis-Tris gels (Invitrogen), transferred to PVDF, and blocked in 10% non-fat milk in TBST for 1 hour. Membranes were then incubated with primary antibodies obtained from Cell Signaling at 1:1000 in 2% non-fat milk in TBST overnight at 4°C. Membranes were washed in TBST, incubated with the appropriate HRP-conjugated secondary antibody (Thermo-Scientific) for 1 hour, washed in TBST, and visualized using ECL-Plus (Amersham) and X-Ray film (Thermo-Scientific).

### In vivo studies

All animal experiments were conducted in accordance with the Institutional Animal Care and Use Committee. Athymic female nude mice (Charles River Laboratories) were inoculated with 5X10^6^ of cancer cells in each rear flank as a 200 μL suspension of 1:1 Matrigel (BD):PBS. Treatment was administered by intraperitoneal injections at a total volume of 200 μL in DMSO. For tissue analysis, tissue was harvested from euthanized mice and fixed in 4% paraformaldehyde in PBS for 48 hours. Tissue was paraffin-embedded and sectioned by the Histology Core Facility at Penn State Hershey Medical Center. H&E staining (Daiko) and TUNEL staining (Millipore) were carried out according to the manufacturer’s protocols. TUNEL assessment was carried out by manual counting of positive cells in ten random fields of view. Bioluminescent imaging and immunohistochemistry for TRAIL expression was performed as previously described [26].

### Microscopy

Cells were grown in chamber slides under sterile conditions as indicated. At end point, cells were fixed using BD Cytofix/Cytoperm according to the manufacturer’s protocol. Following fixation, cells were incubated with an anti-gamma H2AX antibody (Calbiochem DR1017) at 1:200 for 2 hours, rinsed, incubated with an Alexafluor 488 secondary antibody at 1:250 for 30 minutes, stained with Hoechst 33342 at 1 μg/mL for 5 minutes, rinsed, and imaged.

## Abbreviations

DR4: Death receptor 4
DR5: Death receptor 5
ERK: Extracellular signal-regulated kinases
H&E: Hematoxylin and eosin
NCI: National Cancer Institute
TRAIL: TNF-related apoptosis-inducing ligand
TIC: TRAIL-inducing compound
